# Impact of Isosorbide Diesters from Coconut and Sunflower Fatty Acids on Pediatric Atopic Dermatitis and the Skin Microbiome: A Randomized, Double-Blind, Vehicle-Controlled Trial

**DOI:** 10.3390/jcm15020829

**Published:** 2026-01-20

**Authors:** Zill-e-huma Khan, Ajay S. Dulai, Alanna O’Neill, Mildred Min, Joie Lee, Caitlin Dion, Nasima Afzal, Ratan K. Chaudhuri, Andy Lee, Raja K. Sivamani

**Affiliations:** 1 Integrative Research Institute, Sacramento, CA 95819, USA; 2 Integrative Skin Science and Research, Sacramento, CA 95815, USA; 3 School of Medicine, Creighton University, Phoenix, AZ 85012, USA; 4 College of Medicine, California Northstate University, Elk Grove, CA 95757, USA; 5 College of Nursing, California State University, Sacramento, CA 95819, USA; 6 Pacific Union College, Angwin, CA 94508, USA; 7 Sytheon (Now Part of Hallstar), Parsippany, NJ 07054, USA; 8 Pacific Skin Institute, Sacramento, CA 95815, USA; 9 School of Medicine, University of California, Davis, Sacramento, CA 95817, USA

**Keywords:** atopic dermatitis, coconut oil, sunflower seed oil, isosorbide diesters, pediatric, skin microbiome

## Abstract

**Background/Objectives**: Topical application of isosorbide diesters (IDEAS) derived from coconut and sunflower seed oil improve atopic dermatitis (AD) and reduce topical steroid use in adults. This randomized, double-blind, vehicle-controlled trial evaluates topical IDEAS (isosorbide diesters) with colloidal oatmeal for pediatric AD. **Methods**: Subjects aged 2–17 with mild to moderate AD applied either colloidal oatmeal cream or colloidal oatmeal cream with IDEAS daily. Hydrocortisone 2.5% was used as needed. AD severity, itch, sleep, steroid use, and microbiome data were collected at baseline, week 4, and week 8. **Results**: More participants in the IDEAS group compared to the control group achieved EASI 50 (81.0% vs. 56.3%, *p* = 0.10) and EASI 75 (42.9% vs. 18.8%, *p* = 0.12) and achieved a 4-point reduction in subjective itch at week 4 (45.5% vs. 6.3%, *p* = 0.0085) and week 8 (42.9% vs. 12.5%, *p* = 0.045). Use of topical steroids was lower in the IDEAS group (3.4 g vs. 13.3 g, *p* = 0.012) and the relative abundance of *Staphylococcus aureus* was reduced after 8 weeks. **Conclusions**: The addition of IDEAS to colloidal lotion improved AD, improved itch, reduced the use of topical steroids, and reduced the relative abundance of *S. aureus* in the skin microbiome.

## 1. Introduction

Atopic dermatitis (AD) is a common inflammatory skin condition which often presents in infancy with dry skin, lichenification, and significant pruritus. Affecting 10% to 30% of children in developed countries, AD reduces quality of life by causing painful fissures and itchiness leading to excoriation, reduces quality of sleep, and impacts social life due to the visibility of the disease [1]. Typically, topical corticosteroids (TCS) are used as first-line therapy [2]. However, many parents are apprehensive about using TCS because of its potential association skin atrophy, pain or stinging, fissures, adrenal suppression in children, and topical steroid withdrawal [3]. Accordingly, there is increased interest in alternatives to steroids including botanically derived ingredients.

Sunflower seed oil (SSO) is derived from the *Helianthus annus* plant and is high in linoleic acid (LA) which has previously demonstrated benefit to the skin barrier by increasing peroxisome proliferator-activated receptor alpha (PPAR-a) and reducing inflammation [4]. Previous studies have demonstrated that SSO was effective in improving the skin barrier and decreasing TEWL [5].

Coconut oil is extracted from a mature *Cocos nucifera* plant. It has previously demonstrated therapeutic effects as an emollient in treating AD and has demonstrated antimicrobial and anti-inflammatory properties [6]. In both adult and pediatric AD populations, topical coconut oil was shown to improve the severity of AD in comparison to topical mineral oil [7]. Furthermore, virgin coconut oil has been shown to reduce the presence of *Staphylococcus aureus* on the surface of adults with AD [8].

Isosorbide diesters (IDEAS) may have properties that support the skin barrier and health [9]. The results suggest that effects of topically applied fatty acid diesters are not limited to barrier repair but may include inhibition of inflammatory cascades that amplify pruritus and cutaneous eruption. IDL (isosorbide di-(linoleate/oleate)) consists of fatty acid esters derived from sunflower oil, comprising around 70% linoleate and 15% oleate. IDL was shown in a study to have pro-differentiation effects and increased abundance of filaggrin and involucrin, key barrier proteins [10]. (IDC (isosorbide dicaprylate) was derived from coconut fatty acids consisting of mono- and di-caprylic acid esters of isosorbide. IDC was shown to improve skin hydration over glycerol [11]. These results demonstrate potential mechanisms of isosorbide diesters as promising agents for improving barrier function.

The selection of IDL derived from sunflower seed oil and IDC was informed by prior studies demonstrating complementary and synergistic effects on epidermal barrier repair, hydration, and inflammation, processes central to the pathophysiology of inflammatory and xerotic skin conditions [5,12,13,14,15]. IDL has been shown to restore epidermal differentiation and barrier integrity while suppressing inflammatory gene expression, whereas IDC primarily enhances skin hydration and supports early barrier recovery [10,11]. When combined, these esters have demonstrated synergistic anti-inflammatory activity, including enhanced repression of TNF-α–responsive and pruritus-associated mediators, supporting their concurrent evaluation as a barrier-active therapeutic strategy [9].

Colloidal oatmeal is a well-established therapeutic agent known to improve AD severity, normalize skin pH, enhance skin barrier function, and reduce Staphylococcus colonization [16]. These benefits are largely attributed to avenanthramides, bioactive polyphenols in oats, which are thought to inhibit nuclear factor-kappa B (NF-κB) and suppress the release of proinflammatory cytokines [17]. A prior study in adults with AD showed that IDEAS, along with colloidal oatmeal, improved clinical outcomes and reduced topical steroid use, [18] and this investigation assesses these endpoints in a pediatric population.

## 2. Materials and Methods

### 2.1. Study Design, Recruitment and Randomization

This is an 8-week double-blind vehicle-controlled clinical trial that was approved by the Allendale Institutional Review Board (Protocol# IDEAS_PEDS_AD). The participants were recruited directly from local medical clinics and through social media advertisements. Males and females aged 2–17 years were recruited and screened for eligibility, and all study procedures were performed at Integrative Skin Science and Research in Sacramento, CA, USA.

**Figure 1:**
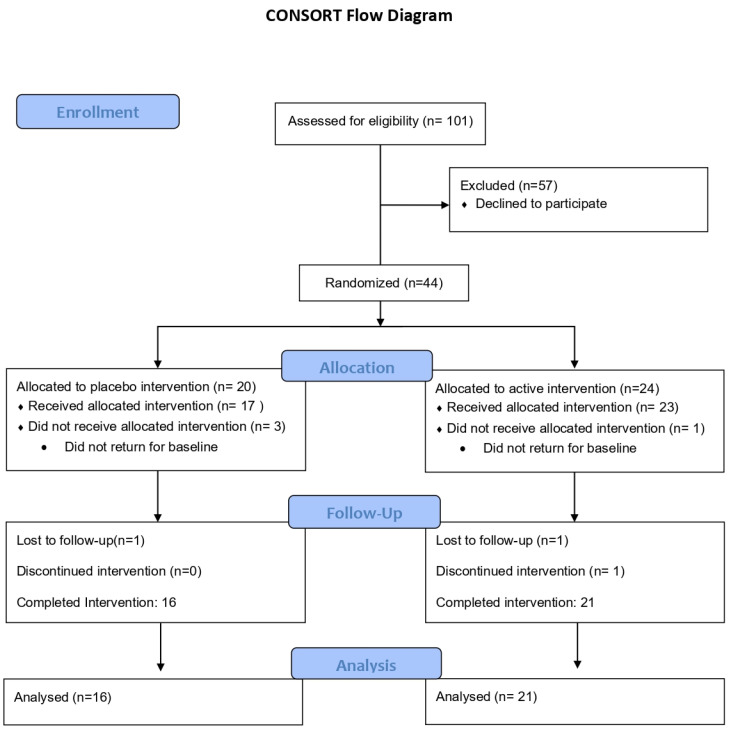
CONSORT diagram.

Forty-four participants met the eligibility criteria and were randomized and assigned into 2 treatment groups. Group assignment was conducted a priori by a clinical research coordinator using a computer-based randomization generator and sequentially drawn blinded sealed envelopes. The IDEAS (Isosorbide Diesters) group (n = 24) received IDEAS (with 0.1% colloidal oatmeal lotion), and the vehicle group received the same 0.1% colloidal oatmeal lotion without the IDEAS in opaque tubes that were coded to maintain the blind. Both groups were prescribed topical hydrocortisone 2.5% and a standardized body soap. Both groups were instructed to apply the lotions to affected areas of the body daily for 8 weeks. The participants were instructed to only use the steroid cream as needed for any rash or breakthrough flares. Use of the topical steroids was monitored by weighing the tubes at each visit. The subjects were evaluated at screening, baseline, week 1, week 4 and week 8. A total of 38 participants completed the study per-protocol (Figure 1). The assessors, coordinators, and analysts were blinded to the allocation.

### 2.2. Inclusion and Exclusion Criteria

Male and female subjects between the ages of 2 and 17 with at least a 6-month history of diagnosed active atopic dermatitis were recruited for the study (Table 1).

A validated Investigator Global Assessment-Atopic Dermatitis (vIGA-AD) score of 2 or 3 (mild to moderate) and an EASI score ≥ 5 were necessary at baseline. Pregnant women, prisoners, those unable to consent, and individuals with a known allergy to IDEAS, coconut oil, or sunflower oil and those who had dermatitis solely on their hands and/or feet were excluded from the study. Subjects were excluded if they were currently on any systemic therapy or needed systemic therapy in the opinion of the investigator. The subjects were required to discontinue select topical and systemic therapies prior to baseline. A washout period of two weeks was required for topical JAK inhibitors, topical calcineurin inhibitors, and crisaborole, and one week for topical corticosteroids. Systemic therapies, including cyclosporine, systemic corticosteroids, methotrexate, and systemic JAK inhibitors, required a one-month washout period, while biologic therapies (tralokinumab or dupilumab) required a two-month washout period. Subjects who refused to undergo a washout prior to starting intervention were excluded from the study. Additionally, subjects were excluded if they had ongoing active infection.

### 2.3. Measurements of the Biophysical Properties of the Skin

Measurements were collected at the initial baseline visit, week 1, week 4, and week 8 of applying study products. All measurements were collected after subjects spent fifteen minutes in a climate-controlled room to ensure adjustment to ambient conditions. The following biophysical properties of the predetermined diseased areas were measured: TEWL using the Vapometer^®^ (Delfin Technologies Ltd., Kuopio, Finland) and skin hydration level by the MoistureMeterSC^TM^ (Delfin Technologies Ltd., Kuopio, Finland).

### 2.4. Assessments of Eczema Area Severity Index (EASI) Score

Each subject’s EASI score was assessed at all study visits by recording the erythema, papulation, excoriation, and lichenification at each of the four regions of the body, including the head and neck areas ,the trunk, and the upper and lower limbs, along with the percentage of affected skin in each body area. The proportion of patients that achieved a 50% improvement (EASI50) or a 75% improvement (EASI75) was noted.

### 2.5. Subjective Reporting of Itch and Sleeplessness on Visual Analog Scale

All subjects were asked to report their level of itching and sleeplessness at every visit on a validated numerical rating system with a 10 cm line by making a tick mark on the line where they felt best represented their symptom severity.

### 2.6. Materials

Isosorbide di-(linoleate/oleate) (IDL) and Isosorbide dicaprylate (IDC) are commercially available from Sytheon (Parsippany, NJ, USA) under the trade names HydraSynol^®^ IDL (INCI: Isosorbide Disunflowerseedate; CAS no. 1818326-42-9) and HydraSynol^®^ DOI (INCI: Isosorbide Dicaprylate; CAS no. 64896-70-4), respectively. Composition of IDL consisted of approximately 70% linoleate and 15% oleate with other minor fatty acid esters. Composition of IDC consisted of >99% mono- and di-caprylic acid esters of Isosorbide with diester content > 95%. Colloidal oatmeal (Colloidal Oatmeal USP/NF, CAS# 134134-86-4) was obtained from Charkit Chemical Company (Norwalk, CT, USA). Key components used in the IDEAS o/w lotion are 4% IDL, 4% IDC and 0.1% Colloidal oatmeal. The vehicle lotion contained no IDL or IDC but contained 0.1% Colloidal oatmeal.

### 2.7. Skin Microbiome Analyses

#### 2.7.1. Bacteria 16S rRNA Gene Variable Regions 1-8 (bV18-A) Sequencing

DNA extraction, library preparation, bacteria 16S rRNA gene variable regions 1–8 (bV18-A), and bioinformatic processing have previously been described in the report CP06164_report.pdf (dated 10 June 2025). The data used in this report has not undergone further processing. Some species in the database share identical V1V8 sequences, making it impossible to distinguish between them. In such cases, all species are included in the taxonomic abundance matrices, with read counts distributed among the species sharing identical DNA sequences.

##### DNA Extraction and Quantification

DNA from samples was pre-treated using MetaPolyzyme (Sigma-Aldrich, St. Louis, MO, USA) and isolated using the QIAGEN DNeasy PowerWater Kit (Redwood City, CA, USA), according to the manufacturer’s protocol. DNA samples were quantified using Qubit Flex fluorometer and Qubit™ dsDNA HS Assay Kit (Thermofisher Scientific, West Sacramento, CA, USA).

##### Library Preparation and Sequencing

Amplicon libraries for the bacteria 16S rRNA gene variable regions 1–8 (bV18-A were prepared using a custom protocol. Up to 25 ng of extracted DNA was used as template for PCR amplification, and each PCR reaction (50 μL) contained 0.2 mM dNTP mix, 0.01 units of Platinum SuperFi DNA Polymerase (Thermo Fisher Scientific, USA), and 500 nM of each forward and reverse primer in the supplied SuperFI Buffer. PCR was performed with the following program: Initial denaturation at 98 °C for 3 min, 25 cycles of amplification (98 °C for 30 s, 62 °C for 20 s, 72 °C for 2 min) and a final elongation at 72 °C for 5 min. The forward and reverse primers used include custom 24 nt barcode sequences followed by the sequences targeting bV18-A: [8F] AGRGTTYGATYMTGGCTCAG and [1391R] GACGGGCGGTGWGTRCA [19].

The resulting amplicon libraries were purified using the standard protocol for CleanNGS SPRI beads (CleanNA, Waddinxveen, Netherlands) with a bead to sample ratio of 3:5. DNA was eluted in 25 μL of nuclease free water (Qiagen, Redwood City, CA, USA). Sequencing libraries were prepared from the purified amplicon libraries using the SQK-LSK114 kit (Oxford Nanopore Technologies, Oxford, UK) according to manufacturer protocol with the following modifications: 500 ng total DNA was used as input, and CleanNGS SPRI beads for library clean-up steps. DNA concentration was measured using Qubit dsDNA HS Assay kit (Thermo Fisher Scientific, USA). Gel electrophoresis using Tapestation 2200 and D1000/High sensitivity D1000 screentapes (Agilent, Santa Clara, CA, USA) was used to validate product size and purity of a subset of amplicon libraries.

The resulting sequencing library was loaded onto a PromethION R10.4.1 flow cell and sequenced using the MinKNOW 24.06.15 software (Oxford Nanopore Technologies, UK). Reads were basecalled and demultiplexed with MinKNOW Dorado 7.4.14 using the super accurate basecalling algorithm (config r10.4.1_400bps_sup.cfg) and custom barcodes.

#### 2.7.2. Bioinformatics Analysis Methods—Bacteria 16S rRNA Gene Variable Regions 1-8 (bV18-A) Sequencing

The sequencing reads in the demultiplexed and basecalled fastq files were filtered for length (320–2000 bp) and quality (phred score > 17) using a local implementation of filtlong v0.2.1 with the settings –min_length 320 –max_length 2000 –min_mean_q 98. The filtered reads were mapped to the species-representative (reps) 16S rRNA (SSU) sequences from the GTDB release 220 database with minimap2 v2.24-r1122 using the -ax map-ont command [19] and downstream processing using samtools v1.14 [20]. The merged data set for bacteria and archaea entries (bac120_ssu_reps_r220.fna and ar53_ssu_reps_r220.fna) was filtered for archaea SSU > 450 bp, and bacteria SSU > 650 bp. Mapping results were filtered such that query sequence length relative to alignment length deviated < 5%. It is noteworthy that low-abundant OTUs, making up <0.1% of the total mapped reads within each sample, were disregarded as a data denoising step. Additionally, each individual read must be observed at least 10 times within a sample to pass the denoising step. Further bioinformatic processing was performed via RStudio IDE (2024.9.0.375) running R version 4.3.3 (2024-02-29) and using the R packages: ampvis2 (2.8.9) [21], tidyverse (2.0.0), seqinr (4.2.36), ShortRead (1.60.0) and iNEXT (3.0.1) [22].

#### 2.7.3. Relative Abundance Stacked Bars

Stacked bar figures were generated using phylum-, class-, order-, genus-, and species-level filtered matrices for bacteria. Stacked bar figures for each group were generated using the R package ggpubr [23].

### 2.8. Statistical Analysis

Differences in results were analyzed using a paired Student’s *t*-tests for parametric measures where a *p*-value < 0.05 was deemed statistically significant. A Chi-squared analysis was performed on categorical data where *p* < 0.1 was considered statistically significant. Only data from enrolled subjects that received the study intervention was analyzed on a per-protocol approach.

For the microbiome analyses, the DESeq2 analysis uses a negative binomial distribution model to estimate differential abundance between cohorts based on count data [24]. The algorithm assumes that most features in microbiome data should not vary greatly between conditions, so it preferentially highlights features that (1) are highly expressed/prevalent, (2) have large fold changes in prevalence, and (3) are statistically significantly different.

## 3. Results

### 3.1. Investigator Assessments (EASI and vIGA)

**Figure 2:**
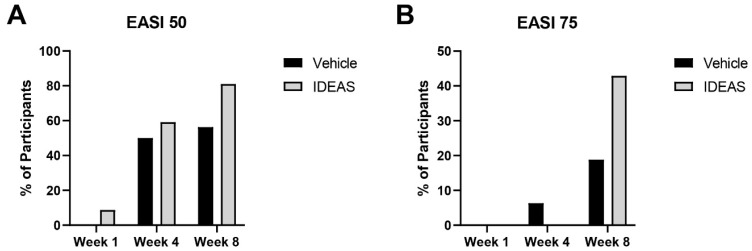
(**A**) Percentage of participants achieving EASI50; (**B**) percentage of participants achieving EASI75.

There was no significant difference between the baseline EASI score for the vehicle (6.6) and IDEAS (7.4) groups (*p* = 0.20). At week 1, there was no significant difference between the percentage of participants achieving EASI50 (*p* = 0.21) in the vehicle (0%) and IDEAS (8.7%) groups, and no participants achieved EASI75 at this visit. At week 4, there was no significant difference in the proportion of individuals achieving EASI50 (*p* = 0.69, vehicle: 50.0%, IDEAS: 59.1%) or EASI75 (*p* = 0.22, vehicle: 6.3%, IDEAS: 0%). At week 8, the IDEAS group had a trend for higher proportion of subjects reaching EASI50 compared to vehicle (*p* = 0.10, vehicle: 56.3%, IDEAS: 81.0%). Additionally, there was a trend for the IDEAS group to achieve EASI75 in comparison to the vehicle (*p* = 0.12, vehicle: 18.8%, IDEAS: 42.9%). The results for week 1, week 4, and week 8 are shown in Figure 2.

At week 4, 12.5% of the vehicle participants and 18.2% of the IDEAS participants achieved IGA success and at week 8, 43.8% of the vehicle participants and 47.6% of the IDEAS participants achieved IGA success. However, there were no statistically significant differences at week 4 or week 8.

### 3.2. Topical Corticosteroid Usage

**Figure 3:**
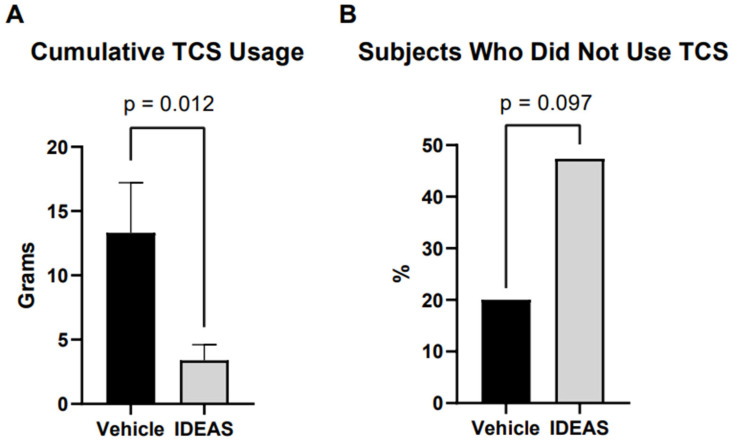
(**A**) Cumulative topical steroid use at week 8; (**B**) proportion of participants who did not use any topical corticosteroids.

The participants in the IDEAS group used significantly less topical steroids throughout the study compared to the vehicle group (*p* = 0.012, 3.4 g vs. 13.3 g) depicted in Figure 3A. When stratifying for patients that never used any topical corticosteroids throughout the entire study, there was a trend for a higher proportion of participants in the IDEAS group compared to the vehicle group (*p* = 0.097, 47.4% vs. 20.0%) depicted in Figure 3B.

### 3.3. Self-Reported Measures (IVAS and DLQI)

**Figure 4:**
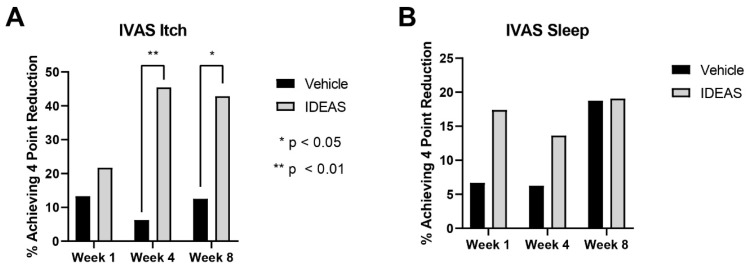
(**A**) Percentage of participants achieving a four-point reduction in subjective IVAS itch scores; (**B**) percentage of participants achieving a four-point reduction in subjective IVAS sleep scores.

There was no significant difference in participants achieving a 4-point reduction in IVAS itch at week 1 (*p* = 0.51, vehicle: 13.3%, IDEAS 21.7%). However, the IDEAS group achieved a 4-point reduction at a higher rate than the vehicle at week 4 (*p* = 0.0085, vehicle: 6.3%, IDEAS: 45.5%) and week 8 (*p* = 0.045, vehicle: 12.5%, IDEAS: 42.9%). This is shown in Figure 4A.

There was no significant difference in the percentage of participants achieving a 4-point reduction in IVAS sleep at week 1 (*p* = 0.34, vehicle: 6.7%, IDEAS: 17.4%), week 4 (*p* = 0.31, vehicle: 6.3%, IDEAS: 13.6%), or week 8 (*p* = 0.98, vehicle: 18.8%, IDEAS: 19.0%) as shown in Figure 4B.

DLQI in the IDEAS group demonstrated a trending absolute reduction at week 4 (−1.14; *p* = 0.077) and a significant reduction at week 8 (−1.24; *p* = 0.035). DLQI in the vehicle group had a trending decrease at week 1 (−2.25; *p* = 0.068), but no significant reduction at week 4 (*p* = 0.14) or week 8 (*p* = 0.35).

### 3.4. Skin Biophysical Measurements (Skin Moisture and TEWL)

**Figure 5:**
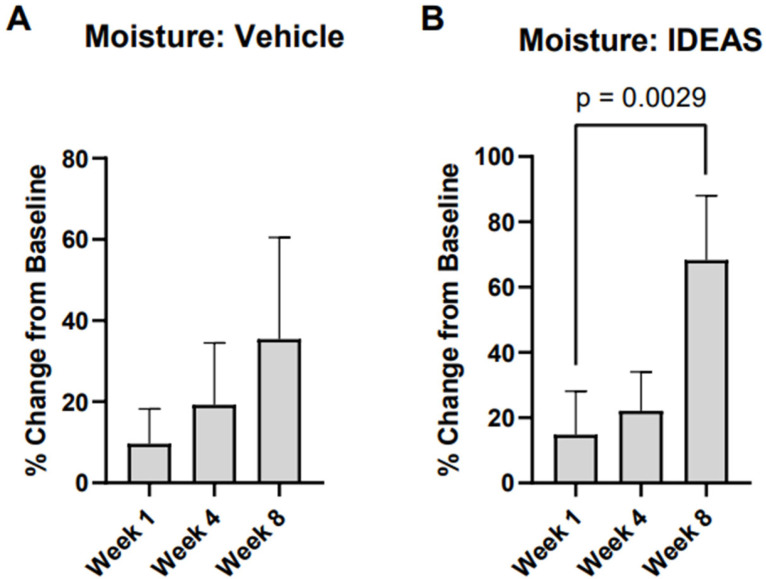
(**A**) Percentage increase in skin hydration in vehicle; (**B**) percentage increase in skin hydration in IDEAS treatment group.

Skin moisture in the IDEAS group was significantly increased at week 8 (68.3%; *p* = 0.0029) while there was no significant change in the skin moisturization in the vehicle group (*p* > 0.05) (Figure 5). Neither the vehicle nor IDEAS group demonstrated significant changes in TEWL at week 1, week 4, or week 8 (*p* > 0.05).

### 3.5. Skin Microbiome Shifts

**Figure 6:**
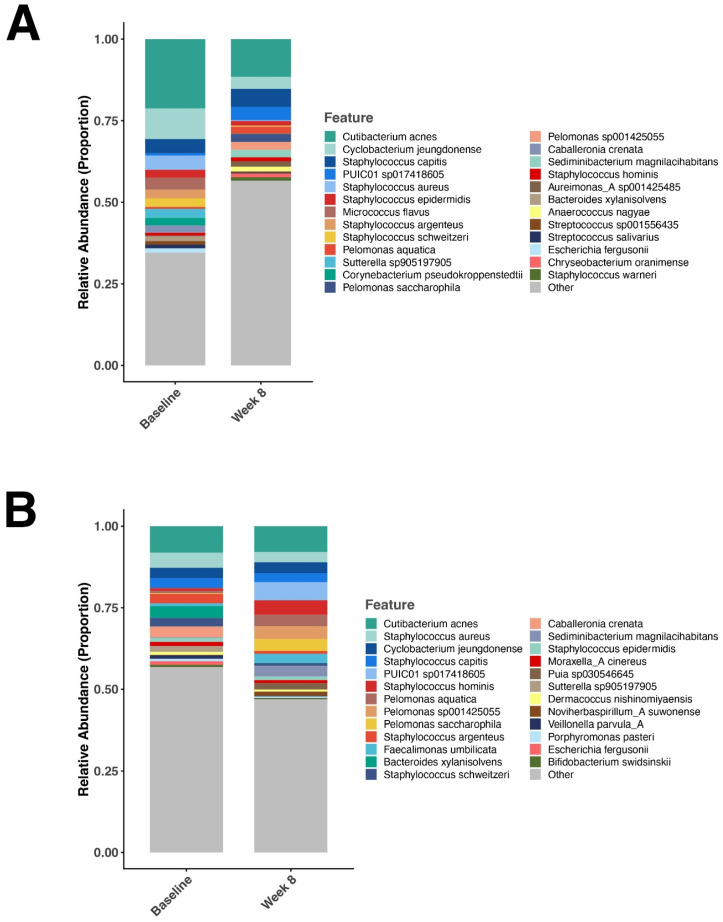
Changes in the skin microbiome relative abundance over time. (**A**) Relative abundance of the most prevalent bacterial taxa in the IDEAS-treated group at baseline and week 8. (**B**) Relative abundance of the most prevalent bacterial taxa in the vehicle-treated group at baseline and week 8. Stacked bar plots represent the proportional composition of bacterial taxa derived from 16S rRNA gene sequencing, with each color corresponding to an individual taxon as indicated in the legend.

**Figure 7:**
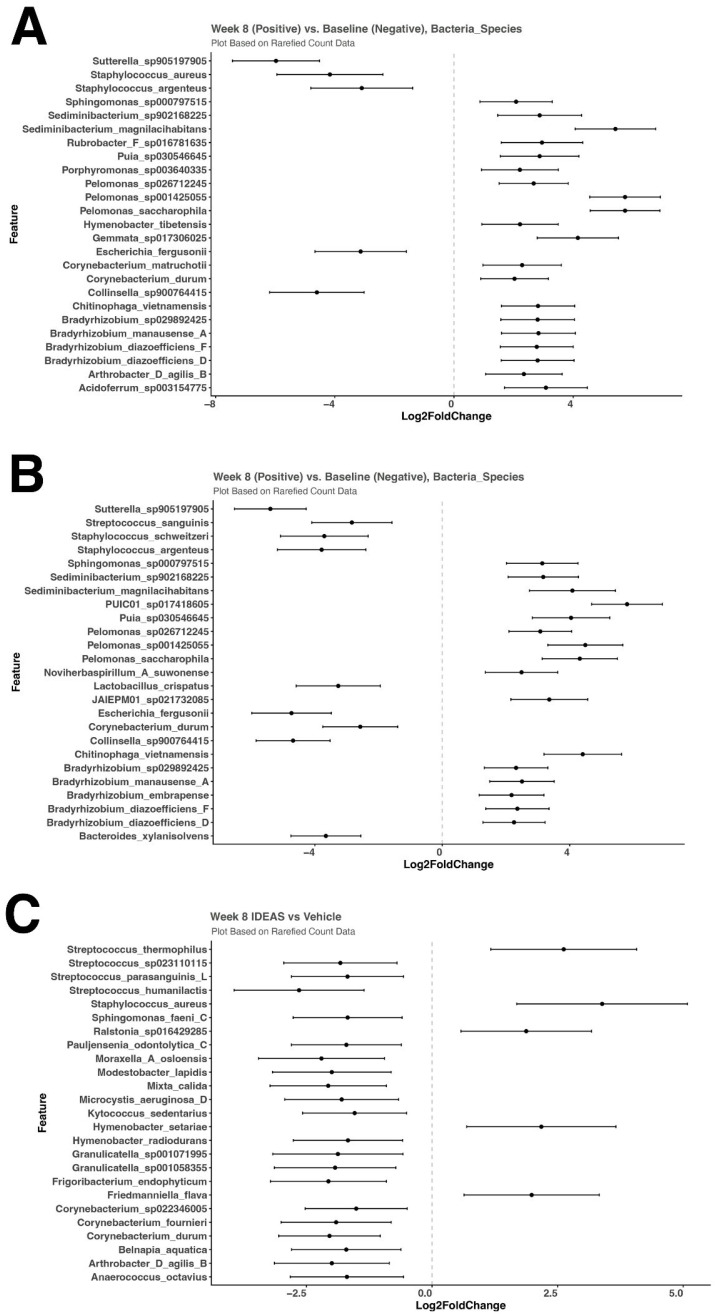
Differential abundance of the skin microbiome between cohorts. (**A**) Differential expressions of different bacterial species are depicted with baseline on the left and week 8 on the right within the IDEAS treated group. (**B**) Differential expressions of different bacterial species are depicted with baseline on the left and week 8 on the right within the vehicle treated group. (**C**) Comparative differential expressions of the skin bacterial species are shown for IDEAS (left) and vehicle (right).

There were no differences in the relative abundance of skin microbiome diversity at baseline. The relative abundance of *Staphylococcus aureus* was reduced when comparing 8 weeks to baseline in the IDEAS group shown as a stacked bar in Figure 6A. However, there was no change in the levels of *S. aureus* in the vehicle treated group after 8 weeks (Figure 6B). A comparative analysis of the skin microbiome at week 8 versus baseline was conducted and bacterial species that had a log2fold change were documented. Figure 7A shows that *S. aureus* was reduced in the IDEAS group at week 8; however, no significant change was seen in the vehicle group (Figure 7B). When comparing the abundance of *S. aureus* in the two groups at the end of 8 weeks, *S. aureus* elevated in the vehicle treated skin compared to the IDEAS treated skin (Figure 7C).

## 4. Discussion

Our findings suggest that the IDEAS group performed better than the vehicle group in both improving AD and in reducing the total use of topical steroids. Additionally, the IDEAS group showed improvements in itch, sleep, and improvement in the quality of life. Our findings are similar to those of previous studies in adults [18]; however, the reduction in topical steroid use was more pronounced in the present study, with a 3.9-fold difference observed at week 8. Concern for the use of topical steroid are typically high among patients with AD, especially among parents of children with AD, with nearly two-thirds expressing discomfort [25]. Therefore, the decrease in the use of topical steroids may have relevance when discussing moisturizer options in the clinical setting.

The efficacy of IDEAS may be attributed to the skin supportive properties of SSO and coconut oil. SSO is rich in LA, which activates PPAR-alpha and maintains the skin barrier by regulating cell proliferation, accelerating repair, and modulating inflammation [4]. LA can also produce anti-inflammatory substances such as prostaglandin E1 and thromboxane A1 [26]. These capabilities provide potential mechanisms by which the use of IDEAS may reduce local inflammation. Several components of IDEAS have previously demonstrated clinical benefits in improving skin hydration and barrier function [10]. Our results agree with previous results in that skin hydration was increased selectively in the IDEAS group (Figure 5).

IDEAS have anti-inflammatory properties in tissue culture and ex vivo studies. Using reconstructed human epidermis models of AD and ex vivo skin cultures stimulated with type 2 proinflammatory cytokines (IL-4, IL-13, TNF-α, and IL-31), the researchers found that co-treatment with IDL and IDC prevented cytokine-induced epidermal barrier disruption [9]. The combination of IDL and IDC also synergistically downregulated inflammatory mediators such as IL1B and ITGA5, as well as the neurogenic pruritus mediators TRPA1 and TRPV3. Treatment of cytokine-treated explants with IDL or IDC also decreased TSLP production. These authors also proposed that the IDL + IDC anti-inflammatory response more closely resembles that of a calcineurin inhibitor rather than corticosteroid. In our study, several clinical outcomes suggest a potential anti-inflammatory effect associated with IDEAS use. Participants in the IDEAS group demonstrated a higher proportion achieving EASI50 and EASI75 at week 8 compared with vehicle, including a 42.9% EASI75 response, although these differences did not reach statistical significance, likely due to limited sample size. Additionally, subjects treated with IDEAS used fewer grams of topical corticosteroids over the study period, with a greater proportion of participants avoiding corticosteroid use entirely, approaching statistical significance. Pruritus outcomes further support this observation, as a significantly higher proportion of IDEAS-treated subjects achieved a ≥4-point reduction in itch at weeks 4 and 8. Collectively, these trends suggest that IDEAS may contribute to reduced skin inflammation and symptom burden beyond just skin hydration and barrier, warranting further investigation in larger powered studies.

The modest EASI50 and EASI75 response rates should be interpreted in the context of permitted background hydrocortisone use, which may have reduced the ability to detect larger between-group differences. Despite this, the IDEAS group demonstrated favorable trends in disease reduction, reduced topical corticosteroid use, and significant improvements in pruritus, supporting a potential benefit beyond standard care alone. These findings suggest that IDEAS may serve as a well-tolerated adjunctive or alternative topical option, particularly for pediatric patients who require steroid-sparing approaches, have limited tolerance to conventional therapies, or prefer formulations with naturally derived ingredients.

The improvement in itch, sleep, and DLQI have important implications. Children with AD are particularly impacted by AD as uncontrolled itch often leads to poor sleep, which can result in more mental health issues such as depression and anxiety [27]. Although neither depression nor anxiety were assessed in this study, it would be warranted to evaluate these factors in future long-term studies that utilize IDEAS as a topical emollient. Parents of children with AD are estimated to lose 40 min to 2.5 h of sleep per night [28] and parental quality of life is also closely tied to itch and sleep in pediatric AD [29]. Therefore, the improvement in sleep and itch may also warrant evaluation of parental sleep and quality of life in future clinical studies.

Our results suggest that topical application of IDEAS reduces the relative abundance of *S. aureus* after 8 weeks of topical application whereas there was no change in the vehicle group. This suggests that topical IDEAS may induce a beneficial shift in the skin microbiome in AD, where S. aureus overabundance is commonly associated with AD activity [30]. Furthermore, our results are in agreement with our previous study in adults that also showed that the relative abundance for *S. aureus* was decreased in response to topical application of the IDEAS based moisturizer [18]. It is unclear whether shifts in *S. aureus* may influence AD severity; however, a previous examination of *S. aureus* relative abundance indicated that such changes precede either flares or resolution of AD [30]. Future kinetic studies may be warranted to clarify whether shifts in *S. aureus* influence or react to changes in atopic dermatitis severity.

This clinical study has several limitations. This intervention period was limited to 8 weeks; therefore, longer-term efficacy remains unknown. In addition, the study population was restricted to individuals with mild-to-moderate AD, and the findings may not be generalizable to patients with severe AD.

In summary, our findings suggest that IDEAS combined with colloidal oatmeal provides enhanced relief for children with AD, alongside normalization of the skin microbiome and a reduction in topical steroid use. Further long-term studies are warranted.

## Figures and Tables

**Table 1:** Subject Demographics.

Characteristic	IDEAS (n = 24)	Control (n = 20)
Age, years, mean ± SD	8.3 ± 4.5	8.8 ± 4.7
Sex, n (%)		
Female	12 (50)	11 (55)
Male	12 (50)	9 (45)
Baseline EASI Score, mean ± SD	7.4 ± 2.4	6.5 ± 1.2
Height, cm, mean ± SD	128.7 ± 26.6	134.2 ± 23.2
Weight, kg, mean ± SD	38.7 ± 22.4	35.8 ± 19.4

## Data Availability

The data supporting the conclusions of this article will be made available by the authors on request.
